# Large B-cell Lymphoma With IRF4 Rearrangement in the Elderly

**DOI:** 10.7759/cureus.102294

**Published:** 2026-01-26

**Authors:** Ryusuke Horaguchi, Shohei Kikuchi, Yoshimi Nabe, Soshi Kisamori, Tomoki Minemura, Takuma Fujihira, Kento Ono, Yusuke Kamihara, Akinori Wada, Tsutomu Sato

**Affiliations:** 1 Department of Hematology, Toyama University Hospital, Toyama, JPN; 2 Department of Internal Medicine, Toyama University Hospital, Toyama, JPN

**Keywords:** diffuse large b-cell lymphoma, elderly lymphoma, head and neck lymphoma, irf4/mum1, large b-cell lymphoma with irf4 rearrangement

## Abstract

Large B‑cell lymphoma with *IRF4* rearrangement (LBCL‑IRF4) predominantly affects children and typically presents with Waldeyer’s ring and/or head and neck involvement, limited stage, and favorable outcomes. Reports in older adults are rare, and comparability to pediatric disease remains uncertain. We describe an elderly LBCL-IRF4 case with a solitary parotid lesion. Histology showed diffuse large B‑cell lymphoma, germinal center B‑cell type, with co‑expression of CD10, BCL6, and IRF4/MUM1. Karyotyping identified t(4;6)(p14;p25) involving 6p25 (*IRF4*), and fluorescence in situ hybridization confirmed interferon regulatory factor 4 (IRF4) rearrangement. The patient achieved an excellent response to R-CHOP therapy, resulting in a complete response. Although clinically similar to pediatric cases, this case involved a non‑immunoglobulin translocation partner, contrasting with the pediatric predominance of immunoglobulin loci. Adult LBCL-IRF4 shows heterogeneity, with some cases resembling pediatric presentations and others diverging. This case underscores the need for further research into age-related clinical and molecular differences in elderly LBCL-IRF4.

## Introduction

Large B-cell lymphoma with IRF4 rearrangement (LBCL-IRF4) was introduced as a provisional entity in the revised 4th edition of the 2017 World Health Organization (WHO) Classification [1]. Subsequently, it was recognized as a definite entity in the 5th edition of the 2022 WHO Classification [2].

LBCL-IRF4 predominantly manifests in children, adolescents, and young adults (AYA) [3,4]. Clinically, it typically arises in Waldeyer’s ring and/or cervical lymph nodes and presents at a limited stage [3,4]. This entity exhibits a favorable prognosis because of its limited-stage presentation and excellent response to chemotherapy [3,4]. The distinctive immunophenotypic profile features strong Interferon regulatory factor 4/Multiple myeloma oncogene 1 (IRF4/MUM1) expression with concurrent BCL6 co-expression [1]. Gene expression profiling and molecular studies have demonstrated that LBCL-IRF4 represents a germinal center-derived B-cell (GCB) lymphoma, with the immunoglobulin heavy chain (IgH) locus serving as the predominant translocation partner of IRF4, consistent with patterns observed across various B-cell neoplasms [3]. Although some studies have documented LBCL-IRF4 in older adults [5-7], key aspects remain uncertain, and it is not yet clear whether elderly patients share the same clinical, pathological, and molecular profiles as pediatric/AYA cases or as the general adult population.

Herein, we present a case of LBCL-IRF4 in an elderly patient. The patient presented with limited‑stage parotid gland involvement and an excellent chemotherapy response, closely aligning with the pediatric pattern. G-banding analysis revealed a non-immunoglobulin translocation partner, suggesting cytogenetic diversity beyond the pediatric/AYA paradigm.

## Case presentation

**Figure 1:**
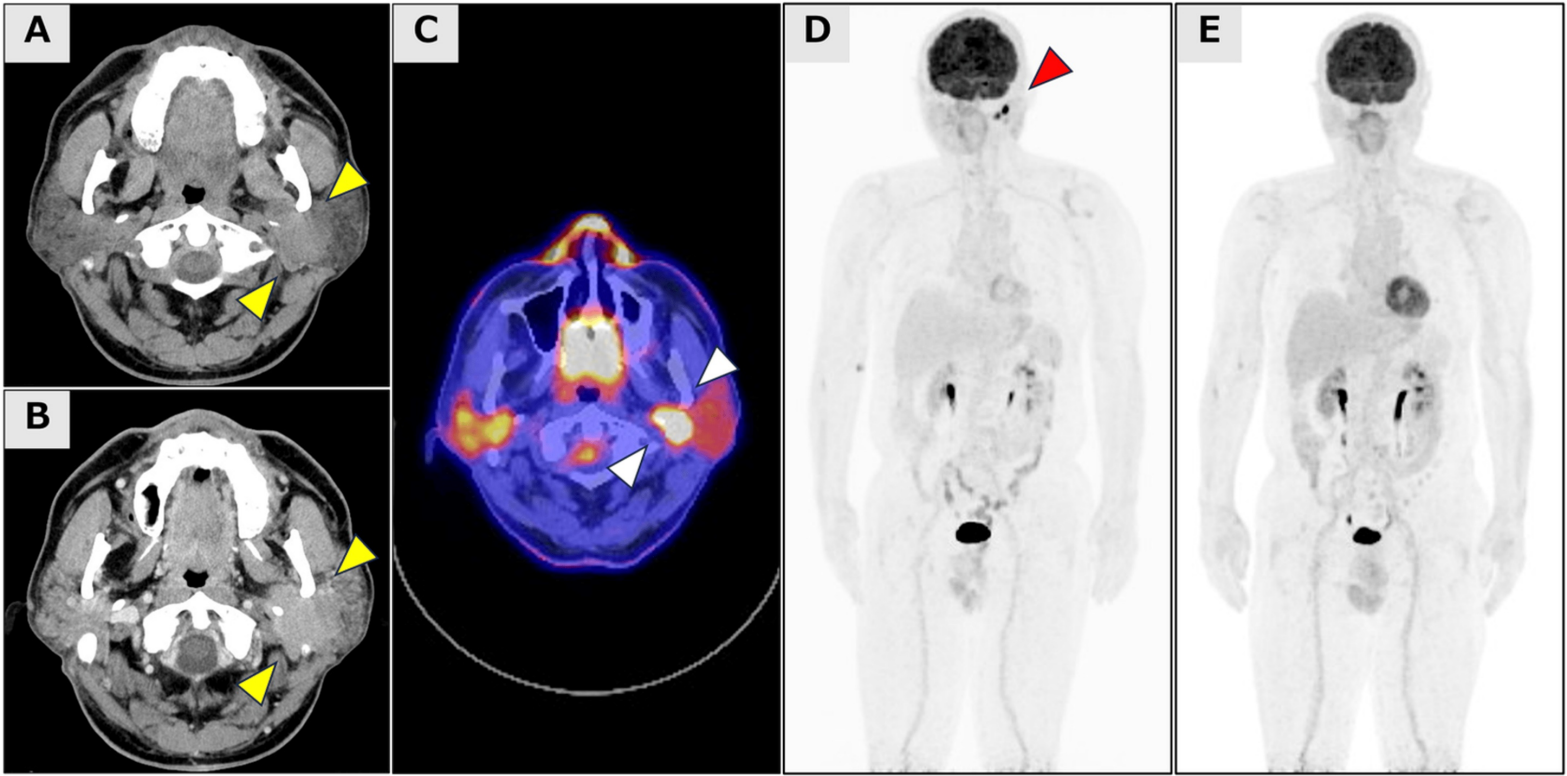
Radiological findings at baseline and post-treatment Axial plain (A) and contrast-enhanced (B) computed tomography (CT) images demonstrate the left parotid tumor with a maximum diameter of approximately 3 cm in the greatest dimension. (yellow arrow) 18-fluorodeoxyglucose positron emission tomography/CT (FDG-PET/CT) demonstrates intense hypermetabolic activity (SUVmax 38.29) corresponding to the left parotid gland lesion (white arrow). (C) Maximum intensity projection (MIP) images of FDG-PET obtained at initial diagnosis (D) and subsequent to the completion of three cycles of R-CHOP therapy (E) demonstrate complete resolution of metabolic activity in the parotid gland lesion (red arrow).

A 71-year-old male presented with progressive enlargement of the left parotid gland region. Upon initial physical examination, palpation revealed notable enlargement without pain or tenderness. CT revealed significant unilateral enlargement of the left parotid gland, measuring 3.1 cm at its greatest dimension (Figure 1A, 1B).

**Figure 2:**
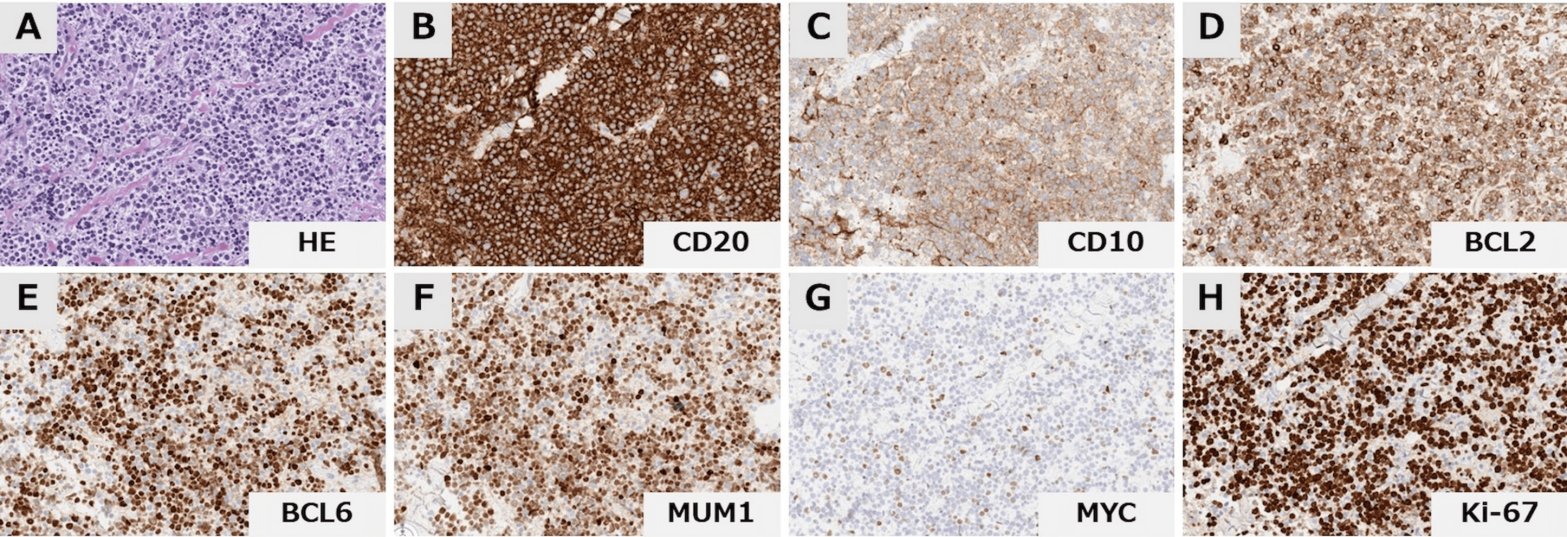
Histopathological findings at diagnosis Histopathological analysis of the parotid gland specimen revealed diffuse proliferation of medium-to-large lymphoma cells. (A) Immunohistochemical analysis demonstrates positivity for CD20, CD10, BCL2, BCL6, and IRF4/MUM1. MYC showed weak positivity in approximately 20% of lymphoma cells, and the Ki-67 labeling index was approximately 80%. (magnification, x40) (B-H)

The patient demonstrated no manifestations of xerostomia or ocular dryness indicative of Sjögren’s syndrome, and serological analysis at admission also revealed negativity for both anti-SS-A and SS-B autoantibodies. An excisional biopsy of the parotid gland revealed diffuse large B‑cell lymphoma (DLBCL) with diffuse proliferation of CD20‑positive medium‑to‑large lymphocytes, without any identifiable low‑grade component (e.g., MALT or follicular lymphoma), consistent with de novo disease (Figure 2). Immunohistochemically, the neoplastic B cells were positive for CD20, CD10, BCL6, and IRF4/MUM1, and negative for CD5 and EBV-encoded RNA (EBER), demonstrating the co-expression of CD10, BCL6, and IRF4/MUM1 and classifying it as GCB-type DLBCL according to the Hans algorithm, although this triple-positive immunophenotype is atypical for conventional GCB-type DLBCL.

**Figure 3:**
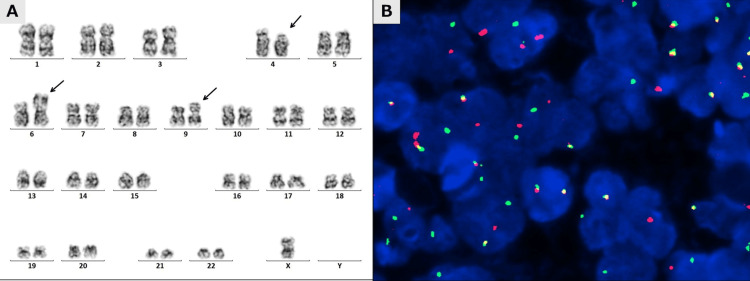
Cytogenetic and molecular cytogenetic analysis demonstrating IRF4 rearrangement A. Chromosome analysis using the G-banded technique revealed a karyotype of 45,X,-Y,t(4;6)(p14;p25), add(9)(p13), demonstrating t(4;6)(p14;p25) involving the 6p25 chromosomal region that harbors the *IRF4* coding gene. B. Representative fluorescence in situ hybridization (FISH) analysis using an *IRF4/DUSP22* break-apart probe shows split signals (green and red) and co-localized signals (yellow). IRF4: Interferon regulatory factor 4; IRF4/DUSP22: Interferon regulatory factor 4/Dual-Specificity Phosphatase 22

G-banding chromosome analysis identified t(4;6)(p14;p25), a translocation involving chromosomal region 6p25, which harbors the *IRF4* coding gene (Figure 3A). Fluorescence in situ hybridization (FISH) analysis using break-apart probes specific for the IRF4 locus revealed a split signal pattern in approximately 65% of examined lymphoma cells, confirming* IRF4* gene rearrangement (Figure 3B).

Whole-body 18-fluorodeoxyglucose positron emission tomography/computed tomography (FDG-PET/CT) revealed a solitary lesion in the left parotid gland with intense hypermetabolic activity (SUVmax 38.29) with no evidence of distant disease (Figure 1C-1E). Further systemic evaluation, including bone marrow examination and gastrointestinal endoscopies, found no evidence of infiltration. Based on the comprehensive clinicopathological evaluation, the patient was diagnosed with limited-stage LBCL-IRF4. The International Prognostic Index (IPI) score was one point, indicating that the patient was at low risk. The patient received three cycles of standard‑dose R‑CHOP (rituximab 375 mg/m², cyclophosphamide 750 mg/m², doxorubicin 50 mg/m², vincristine 1.4 mg/m² [maximum 2.0 mg], and prednisone 100 mg/day for five days) followed by involved‑field radiotherapy. A complete metabolic response, indicative of excellent chemosensitivity, was observed after R-CHOP therapy assessment utilizing FDG-PET/CT. The patient sustained complete remission with no evidence of recurrence over a 30-month follow-up period following completion of the planned consolidative radiation therapy.

## Discussion

Although LBCL-IRF4 predominantly affects the pediatric/AYA populations, accumulating evidence demonstrates its occurrence in older adults. In a cohort of 238 adult DLBCL cases (>20 years), Hesius et al. identified six cases (2.5%) reclassified as LBCL-IRF4 by FISH [7]. Similarly, Streich et al. detected IRF4 rearrangements in 6.7% (2/30) of adult DLBCLs arising in Waldeyer’s ring [8]. Frauenfeld et al. also reported LBCL-IRF4 in 24% (13/55) of cases co-expressing CD10, BCL6, and IRF4/MUM1, a phenotype classified as GCB by the Hans algorithm [5]. Taken together with our elderly patient and previously reported older cases, FISH analysis for IRF4 rearrangement should be strongly considered in adults, especially those with limited head and neck tumors and/or showing co-expression of BCL6 and IRF4/MUM1, with particular attention to the elderly population.

While LBCL‑IRF4 has been reported across the general adult population, evidence specifically addressing elderly patients remains limited. Because LBCL-IRF4 is generally limited-stage and highly chemosensitive with a favorable prognosis, we selected three cycles of R-CHOP followed by involved-field radiotherapy as a regimen with reduced cumulative chemotherapy exposure, aiming to balance disease control with chemotherapy-related toxicity in this elderly patient. Our case showed excellent chemosensitivity, paralleling pediatric patterns. In addition to the favorable biology of LBCL‑IRF4, limited-stage disease and low IPI score likely contributed substantially to the excellent clinical outcome. According to the report of Hesius et al., six cases of LBCL-IRF4 exhibited an age distribution ranging from 26 to 79 years (median: 60 years). Notably, four of these cases (66.7%) demonstrated disease confined to the head and neck region or Waldeyer’s ring. Complete remission was achieved in all five cases that underwent therapeutic intervention [7], demonstrating clinical characteristics comparable to pediatric LBCL-IRF4, consistent with our reported case. Conversely, Berg et al.'s comprehensive analysis of 29 adult LBCL-IRF4 cases demonstrated significant age disparities based on anatomical presentation. The median age of 22 cases with typical sites, such as Waldeyer's ring (16 cases) and cervical lymph nodes (five cases), was 32 years, representing general adult patients, whereas the median age of nine cases with atypical sites, such as skin, soft tissue, and non-cervical lymph nodes, was 68 years, indicating a clear tendency toward atypical site involvement in elderly patients [6].

Most IRF4 translocations partner with immunoglobulin (Ig) loci: Salaverria et al. identified 23 IRF4‑translocated B‑cell lymphomas by FISH, including 17 IGH::IRF4, 2 IGL::IRF4, and 1 IGK::IRF4, supporting Ig loci as predominant partners [3]. In contrast, in the present case, the partner was non‑Ig; because the breakpoint involved 4p14, RHOH, which has been reported to undergo somatic hypermutation in 43% of DLBCL cases [9], was considered a plausible candidate. However, gene‑targeted molecular assays were not performed in this case, which represents a limitation of our analysis and underscores the need for comprehensive molecular profiling in future studies to definitively characterize the IRF4 translocation partner. Consistent with a non‑Ig alternative, Zhang et al. detected RHOH as a novel IRF4 fusion partner in one of three older adult EBV-associated DLBCLs with IRF4 rearrangement [10].

Recent genomic analyses using next-generation sequencing have demonstrated that LBCL-IRF4 has different cell-of-origin (COO) patterns between older adults and pediatric/AYA patients. Pediatric LBCL-IRF4 exhibits a GCB subtype phenotype consistent with the majority of pediatric DLBCLs [11]; however, despite a GCB subtype, it is characterized by frequent mutations in NF-κB pathway genes such as CARD11, CD79B, and MYD88 [12]. These NF-κB pathway mutations are commonly observed in LBCL‑IRF4 across both pediatric and adult cohorts. However, upon stratification of adult IRF4 mutation cases according to GCB/ABC molecular subtypes, patients under 40 years of age or those with GCB molecular subtype exhibited characteristics similar to pediatric LBCL‑IRF4, while those over 40 years of age manifested ABC characteristics absent in pediatric LBCL‑IRF4 [5]. The older‑adult subgroup also shows greater genetic complexity with mutations involving KMT2D, MYD88, and BTG2, suggesting that age- and lineage-related molecular divergence may contribute to the development of atypical cases that differ from pediatric LBCL-IRF4 [5].

## Conclusions

In conclusion, we report a case of LBCL-IRF4 in an elderly patient who, while exhibiting clinical features characteristic of pediatric cases, demonstrated cytogenetic differences in chromosomal analysis. LBCL-IRF4 in older adults likely comprises two distinct groups: cases that genetically and clinically mirror pediatric presentations and those that diverge, warranting comprehensive future investigation. Our case provides insights into this emerging heterogeneity and underscores the importance of proactive IRF4 rearrangement testing in elderly patients and the need for further studies to elucidate the biological basis of these age‑related differences. 
